# Draft Genome Sequences of Eight Bacilli Isolated from an Ancient Roman Amphora

**DOI:** 10.1128/mra.00280-22

**Published:** 2022-05-31

**Authors:** Andrea Colautti, Giuseppe Comi, Emanuele De Paoli, Enrico Peterlunger, Marta Novello, Elena Braidotti, Daniele Pasini, Lucilla Iacumin

**Affiliations:** a Department of Agricultural, Food, Environmental and Animal Science (Di4A), University of Udine, Udine, Italy; b Museo archeologico nazionale di Aquileia e Museo paleocristiano di Aquileia, Direzione regionale musei del Friuli-Venezia Giulia, Aquileia, Udine, Italy; University of Maryland School of Medicine

## Abstract

Paleomicrobiology, the study of ancient microbiological material, allows us to understand different evolutionary phenomena in bacteria. In this study, eight bacilli isolated from an ancient Roman amphora, which dates to the IV to V sec. AD, were sequenced and functionally annotated.

## ANNOUNCEMENT

Bacteria belonging to *Bacillus* spp. are capable of forming spores, specialized cell forms that can withstand adverse environmental conditions and extreme factors, such as temperature, radiation, and chemicals (1), also allowing for survival in a quiescent state for a long time (2). Isolation of these bacterial species from ancient sources has been previously reported, for example from a mummy (3) or ancient soil (4). In this study, bacilli from an ancient Roman amphora were isolated and sequenced. The amphora (IV to V century AD), found in Aquileia (UD-Italy) (45°45'05.9”N 13°21'03.8”E), was found intact and sealed with cementitious compound, thus preventing microbial contamination. It was opened under aseptic conditions in a laminar flow hood and GMP were followed to avoid contaminations. The inner material was sampled using 10 different culture media for bacteria and fungi by serial dilution method and enrichment steps. Growth (7.26±0.09 log colony forming units/g) was observed only in brain heart infusion and plate count agar (Oxoid, Italy) after 48 h at 30°C under aerobic conditions, showing indented, diffuse mucosal colonies, 1 to 2.5 cm in diameter after 48 h at 30°C. The environmental control made using active/passive methods confirmed the absence of *Bacillus* spp. in the laboratory air. Twenty-five colonies present on the counting plates were isolated and examined for their morphological characteristics, which were Gram- and catalase-positive. Preliminary identification was performed by sequencing amplicons obtained using primers P1 and P4 (5), targeting V1 to V3 regions of 16S rDNA. Amplification conditions: final volume 50 μL, 10 mM Tris–HCl, pH 8, KCl 50 mM, MgCl_2_ 1.5 mM, dNTPs 0.2 mM, each primer 0.2 μM, 1.25 U *Taq*-polymerase (Applied Biosystem, I), and 100 ng of DNA. After purification, products were sent to a commercial facility for sequencing (Sanger technology, Eurofins Genomics, Germany). Clones were eliminated by comparing genetic fingerprints (by RAPD, Rep-PCR, SAU-PCR) (6) of isolates and the resulting eight unique individual strains were subjected to whole-genome sequencing. For the sequencing process, each strain was cultured in brain heart infusion broth at 30°C for 48 h. After obtaining the cell pellet by centrifugation for 5 min at 5,000 × *g*, the DNA was extracted with the MagAttract HMW DNA Kit (Qiagen, Germany). The DNA was fragmented by sonication (BioRuptor-Diagenode, Belgium) and Celero DNA-Seq kit (Tecan, Swiss) was used for the preparation of libraries. The size of the individual fragments making up the library was measured using BioAnlayzer 2100 DNA chip electrophoresis (Agilent Technologies, USA) and sequencing was carried out with the MiSeq platform (Illumina, USA) in paired-end mode with reads of 300 bp length. The obtained. fastq files were analyzed and assembled using WGA-LP pipeline (7) with the following tools used in default mode. Raw reads were quality trimmed and deprived of Illumina adapters via Trimmomatic v0.39 (8). FastQC v0.11.9 (9) and Kraken2 v2.0.8-b (10) were used for quality and contamination control. Assembly was carried out using SPAdes v3.15.2 (11). The quality of the final assemblies was evaluated using CheckM v1.1.3 (12), Quast v5.0.2 (13), and SamTools v1.10 (14). Functional annotation was carried out on the genomes using PGAP 2022-04-14.build6021 (15).

### Data availability.

Sequences were deposited in GenBank with PRJNA811801 BioProject accession number. Table 1 reports the GenBank and SRA accession number, the raw reads number, the NCBI taxonomic identification, the isolation source, the sequencing and assembly statistics, and the genome features of strains for each sample.

**TABLE 1:** Statistics of assembled genomes

GeneBank accession no.	SRA accession no.	16S RNA accession no.	Raw reads^*a*^	Strain	Organism name	Source	Coverage	Genome size^*b*^	Scaffolds^*b*^	N50^*b*^	G+C content (%)^*c*^	CDS^c^	tRNAs^*c*^	Completeness (%)^*d*^
JAKXEE000000000	SRR18190504	ON326590	2,727,696	Aquil_B1	*P. simplex*	Amphora	234×	5,649,653	25	910,271	40.2	5,402	81	98.91
JAKXED000000000	SRR18190503	ON326591	717,606	Aquil_B2	*L. fusiformis*	Amphora	75×	4,643,302	34	1,011,198	37.5	4,546	85	99.93
JAKXEC000000000	SRR18190502	ON326592	494,295	Aquil_B3	*B. muralis*	Amphora	46×	5,057,074	38	641,321	41.3	4,721	84	98.77
JAKXEB000000000	SRR18190501	ON326593	1,480,745	Aquil_B4	*B. frigoritolerans*	Amphora	65×	6,677,279	68	317,977	39.5	6,548	92	98.91
JAKXEA000000000	SRR18190500	ON326594	2,409,430	Aquil_B5	*B. muralis*	Amphora	151×	5,067,063	38	641,321	41.3	4,723	84	98.91
JAKXDZ000000000	SRR18190499	ON326595	1,249,575	Aquil_B6	*P. psychrodurans*	Amphora	108×	4,256,356	79	253,084	35.9	4,213	70	100
JAKXDY000000000	SRR18190498	ON326596	1,122,132	Aquil_B7	*B. frigoritolerans*	Amphora	71×	5,521,551	46	613,520	40.3	5,287	84	98.91
JAKXDX000000000	SRR18190497	ON326597	1,742,298	Aquil_B8	*P. simplex*	Amphora	57×	5,654,249	78	193,610	40.2	5437	81	98.91

a Determined using FastQC.

b Determined using Quast.

c Determined using PGAP.

d Determined using CheckM.
